# Evaluation effects of two types of freeway deceleration markings in China

**DOI:** 10.1371/journal.pone.0220811

**Published:** 2019-08-13

**Authors:** Yanqun Yang, Said M. Easa, Xinyi Zheng, Aixiu Hu, Fashui Liu, Meifeng Chen

**Affiliations:** 1 College of Civil Engineering, Fuzhou University, Fuzhou, Fujian, China; 2 Department of Civil Engineering, Ryerson University Toronto, Ontario, Canada; 3 Faculty of Humanities and Social Sciences, Fuzhou University, Fuzhou, Fujian, China; 4 Highway Bureau of Fuzhou, Fuzhou, Fujian, China; Huazhong University of Science and Technology, CHINA

## Abstract

This paper presents an evaluation of the effectiveness of two types of deceleration markings on freeways in China: fishbone-shaped (FS) markings and edge-rate (ER) markings. An actual 4-lane, 6-km long freeway in Province Hebei was created in a driving simulator and used for the experiments. Three scenarios of deceleration markings were adopted: one without markings, one with FS markings, and the third with ER markings. For each scenario, three traffic flow levels were adopted (low, medium, and heavy). The appropriate dimensions of deceleration markings were first established using relevant design guidelines and then used to develop the simulation scenarios using Multigen Creator software. Sixty drivers carried out the simulation experiments with eye tracker equipment. The adequacy of deceleration markings was analyzed with respect to speed, perceived distance, pupil diameter, and geometric parameters. The results showed that both types of deceleration markings made a certain effect on vehicle speed, drivers’visual behavior, and mental characteristics. However, the effect of the FS markings was more pronounced than that of the ER marking. Specifically, the FS markings showed a speed reduction of 12.3 km/h to 15.2 km/h and a perceived distance of 70 m to 90 m, compared with 6.7 km/h to 9.9 km/h and 40 m to 60 m, respectively, for the ER markings. Application comments of the results are provided in the conclusion.

## Introduction

Over the past 30 years of reform and development, China has formed a full coverage transport network. By the end of 2015, China’s total highway traffic length was 4.58 million km, Express way was 123,500 km, ranking first in the world [1]. However, the number of deaths in road traffic accidents in China still rank second in the world although safety management have been manifested in the system of transportation network [2]. The surveys of traffic accidental analysis from the Highway Traffic Police Department in China reported that 38% of traffic accidents were highly related to drivers’ illusion of driving speed [3].

Through the past 50 years research and practical performance, many speed control methods have been developed such as speed limit signs, speed humps and pavement markings. Speed limit signs [4] are less likely to achieve an ideal result with high autonomy requirement and low observance of drivers. The speed hump [5] is a sort of deceleration facilities that based upon the tactile sensation of drivers and made the driver decelerate the vehicle because of vibration and discomfort. However, it demands a high project cost and has a bad effect on the comfortability of driving with noise in tough deceleration. Whereabouts, the visual illusion-based deceleration pavement markings can effectively arouse drivers’notice and induce them to slow down of their own accord [6,7,8]. It makes the drivers feel the acceleration in driving and see the narrowed lanes while run through the deceleration marking section. Accordingly, the effect of speed control and driving comfort is ideal for the use of pavement markings.

Of the several innovative pavement marking patterns to reduce traffic speeds, two kinds of pavement patterns that have been most employed and evaluated are the transverse pattern and chevron pattern [9, 10]. Transverse pattern is firstly proposed going back to the mid-1970s [7]. This pattern consists of a series of stipes or bars placed across the roadway perpendicular to the path of traffic. The first bars or stripes encountered in the pattern are widely spaced, subsequent bars in the pattern are placed closed and closer together. When the pattern works as intended, drivers reduce their speed as rapidly as they should as they proceed through the pattern. The chevron pattern [8] is characterized by a series of chevrons on the pavement surface that are placed progressively closer together. The intent of this pattern is to create illusion that drivers are travelling faster than they really are and to foster the impression that the traffic lanes are narrowing.

Based on the previous literature review in studying the effectiveness of these two pavement marking patterns, general conclusions have been concurred that chevron markings were more relevant to the reduction speed in the role of perception illusion, while transverse markings were more likely to play the role of warning signs and lead to the reduction speed at certain level [7]. In addition, both pavement markings were considered to be employed in the different road conditions. Specifically, chevron markings are applied to the highway or freeway for more effectiveness of reduction speed, while transverse markings are more and less used in the narrow rural road for more warning and alerting effect [11, 12, 13].

Furthermore, many studies discovered that two pavement makings had different effects on speed reduction in terms of driving behavior. Griffin and his colleagues found that horizontal visual markings can reduce the accident rate by 5%~50%, while V-shaped visual deceleration markings 25%~50% [7]. As for the speed-controlling effect discovered that V-shaped visual illusion deceleration markings can effectively reduce the speed, and the effect on trucks is better than that on buses [14]. Voigt found that with the adoption of V-shaped deceleration markings, the average speed in joint section dropped from 55.2km/h to 53.4km/h during the day, while the average speed in the night decreased from 55.5km/h to 51.3km/h [15]. Gates conducted an experiment in regard to the effect of the deceleration marking during different periods. The experiment collected the speed data in the deceleration marking section after 2 weeks, 10 weeks and 6 months respectively, and the result showed the deceleration effect is sharply reduced after 6 months [6]. Review studies found that transverse markings can effectively reduce curve speeds, especially shortly after initial installation. Moreover, such markings was more effective at reducing speed in the lane next to the shoulder than in the median lane [6]. Chevron pavement markings can both produce reasonable reduction in speeds as well as improved driver’s lane position [9, 13]. Such markings are suggested to be used to highlight perceptual cues in highway curves, ramps or tunnels [10, 16].

In China, speed reduction markings are not enforceable because the markings are characterized as non-intrusive speed control devices. Although many Chinese researchers [17, 18, 19, 20, 21, 22, 23] assume that speed reduction marking would make drivers believe that the lane becomes narrower which would generate visual illusion on drivers. However, such assumptions need more theoretical or empirical studies conducted based on the characteristics road specific in China. Over the past few years, Chinese scholars have put forward several common parameters calculation methods of the visual illusion deceleration markings by utilizing drivers’visual illusion theory, and then constructed a driving simulation platform or field study to pick up the combination parameters which receive a better result in deceleration. One study was to use the simulation to analyze the effectiveness of speed reduction markings in downhill section and found that longitudinal speed reduction marking were more effective than transverse markings [12]. Another study focused on the design pattern of city tunnel sidewall and found that the comfort and rationality of the new design patterns has been verified [20]. Recently, a study was to analyze the effects of sidewall markings in highway tunnels by observing how their angles and lengths affect the driver’s speed perception. The results had the reflection of Zöllner illusion suggesting the perception of lane width shrinks may induce deceleration behavior [21]. Recently, Ding et al performed a naturalistic field driving to study the effects of reverse linear perspective of transverse markings on car-following headway in China. The results showed that the car-following time headway was increased after the installation of the reverse linear perspective information [22]. Another Chinese research group [23] recently published the study regarding the effect of transverse markings on left turn direct connectors of urban expressways in China. The results showed that compared to 200m –radius connectors, transverse markings are effective in 300m-radius connectors, particularly in the last 800m of the 300m-radius left turn direct connectors. Apart from these researches in transverse markings studies in China, some other Chinese scholars have made great efforts in applying big data analysis and genetic algorithm model to explore the possible relationship between various road conditions and risk driving behaviors [24, 25]

Although existing studies have analyzed the interaction between the effect of deceleration markings and the visual illusion properties of drivers, many of them used the speed and speed deviation in deceleration marking section as the evaluation indicators, which lacks of the analysis on the eye movement parameters to understand the visual information process on the perception of pavement makings. Eye movement parameters were likely used in the analysis of the perception of hazard cue on road rather than the evaluation of the pavement markings [26, 27, 28] in the previous literatures. Moreover, many studies focused on one single deceleration marking effect, the comparison on the different interaction results between different markings has not been evaluated thoroughly on the basis freeway conditions in China. Therefore, this paper presents an evaluation of the effectiveness of two types of deceleration markings: fishbone-shaped (FS) markings considered as a type of chevron marking and edge rate (ER) markings considered as a type of transverse markings under three traffic flow levels using a driving simulator design. In addition, using eye tracker equipment during the experiments, the range of applications, deceleration effect, visual effect, and security of deceleration markings were analyzed to evaluate the driving effect of two types of marking on the basis of the visual illusion properties of drivers and vehicle’s running performance data, and driver’s fixation/control behavior. The combination of driving simulator and eye-tractor in the study was aimed to complement the fully understanding of the effectiveness of reduction speed markings.

## Design of simulation experiment

### Experimental equipment

**Fig 1:**
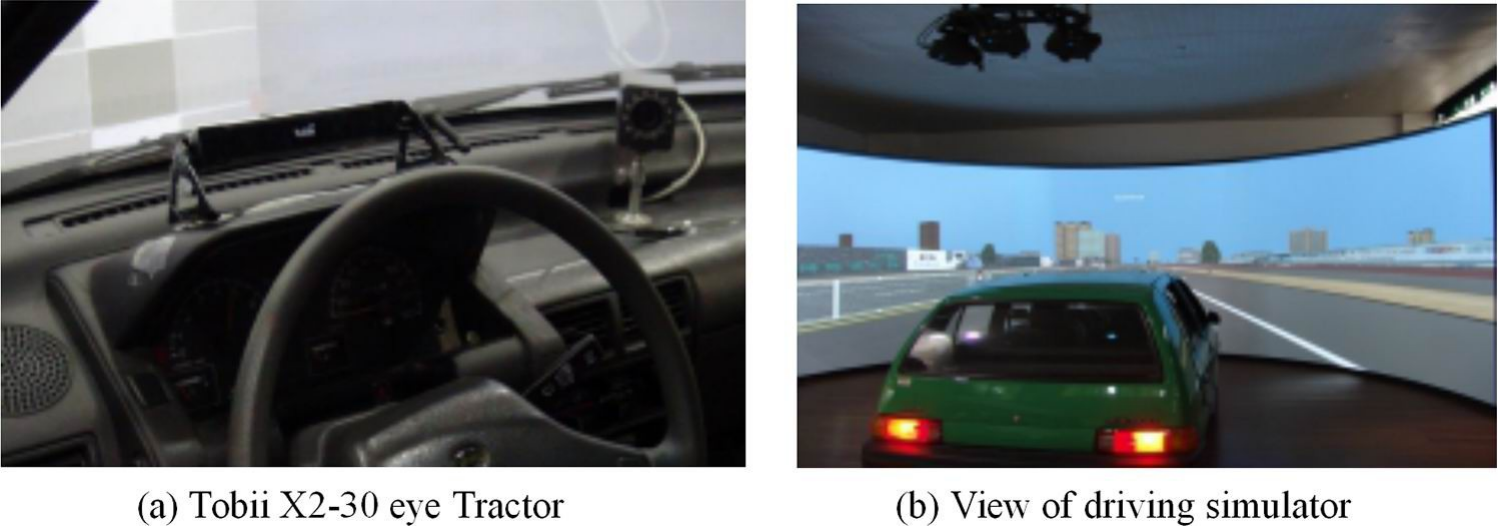
Driving simulator equipment (DSR-1000TS model). (a) Tobii X2-30 eye tractor. (b) View of driving simulator.

The needed information collection devices include vehicle’s driving performance parameters and eye movement parameters. The driving performance parameters were collected using the driving simulator DSR- DSR-1000TS. The road environment is built up by the visual simulation real-time 3D model creation software Creator and Vsdesign, and is projected on the front of the vehicles. The DSR-1000TS driving simulator equipment is shown in Fig 1. The eye movement parameters are collected by eye tracker Tobii X2-30. The collection frequency of the eye tracker is 30Hz, the allowable subjects head movement range is 50×36cm(20×14”), and the distance from the subject to the eye tracker ranged from 40–60. cm. The eye tracker only needs to be calibrated once for the same subject and it will collect and analyze the data by the visual software Tobii Studio.

### Selection of subjects

Sixty drivers with C1 driving licenses were recruited as the experimental subjects. The subjects included 43 males (72%) and 17 females (28%) with ages ranging from 22 to 52, and driving experience ranging from 2 to 10 years. Fatigue driving, drunk driving, and the influence of drugs didn’t exist in the driving test. The distribution of the age categories were as follows: 31 subject (52%) in the range 20–35, 24 subjects (40%) in the range 35–45, and 5 subjects (8%) in the range of over 45. The corrected visual acuity of all drivers was 1.0. All participants were sign the consent form before the experiment and were given 150 Chinese currency (about 25 US dollars) after the experiment fully completed.

### Experimental scenarios

To control relevant variables of the deceleration markings, three experimental scenarios were adopted in this study, as follows:

Scenario 1: This scenario had no deceleration markings.
Scenario 2: This scenario had fishbone deceleration markings.
Scenario 3: This scenario had edge-rate deceleration markings.

The length of each experimental section was about 6 km. For each scenario, three traffic flow levels were adopted (low, medium, and high) to account for the changes in visual and vehicle behavior. The traffic flow included a mix of vehicle types (cars, buses, and trucks), which was simulated as like as the real world driving environment. The traffic mix in vehicles per hour (vph) was selected such that the equivalent traffic flow is in the order of 2000, 3000, and 4000 passenger car units per hour (pcuph) for the three flow levels, reflection of the various road scenario in the real world. Buses and trucks were set to drive on the outside third and fourth lanes, while cars were set to drive in all lanes uniformly and turn right at the interchange with a ratio of 30%. The mix of vehicle types and right-turn ratio at the interchange reflected the data of the Shijiazhuang-Anyang Freeway. The characteristics of the three experimental scenarios are shown in Table 1. Apart from the differences in the type of deceleration markings and traffic flow level, other road elements were the same for the three scenarios.

**Table 1:** Traffic flow levels used for each of the three scenarios.

Traffic Flow Level	Traffic Flow Equivalence, *Q* (pcuph) ^a^	Traffic Composition (vph)		
		Cars	Buses	Trucks
**Low**	2100	800	160	300
**Medium**	3050	1950	140	250
**Heavy**	4015	3200	110	180

^a^ *Q* = one-way traffic flow.

## Construction of simulation scenarios

### Selection of experimental road

In this experiment, an actual freeway in Heibei province is created in the driving simulator. The freeway extends from Huangshi Interchange to the Liushi Village (approximately 6 km from 297+000 to K303+200). The freeway has four lanes (in one direction), each lane is 3.75 meters wide. The alignment is relatively good and does not include any long downgrades, sharp turns, or tunnels. Xiaohe Bridge is 756-m long and its central stake number is K300+413.00. The freeway is straight with a slight fluctuation in vertical alignment. The altitude along the freeway ranges from 40.623 to 47.725 meters with a relative height difference of 7.1 meters. The Shijiazhuang-Anyang freeway section used for the experiments included basic road geometric characteristics, in addition to Xiaohe Bridge and Huangshi Interchange.

### Design of deceleration markings

**Fig 2:**
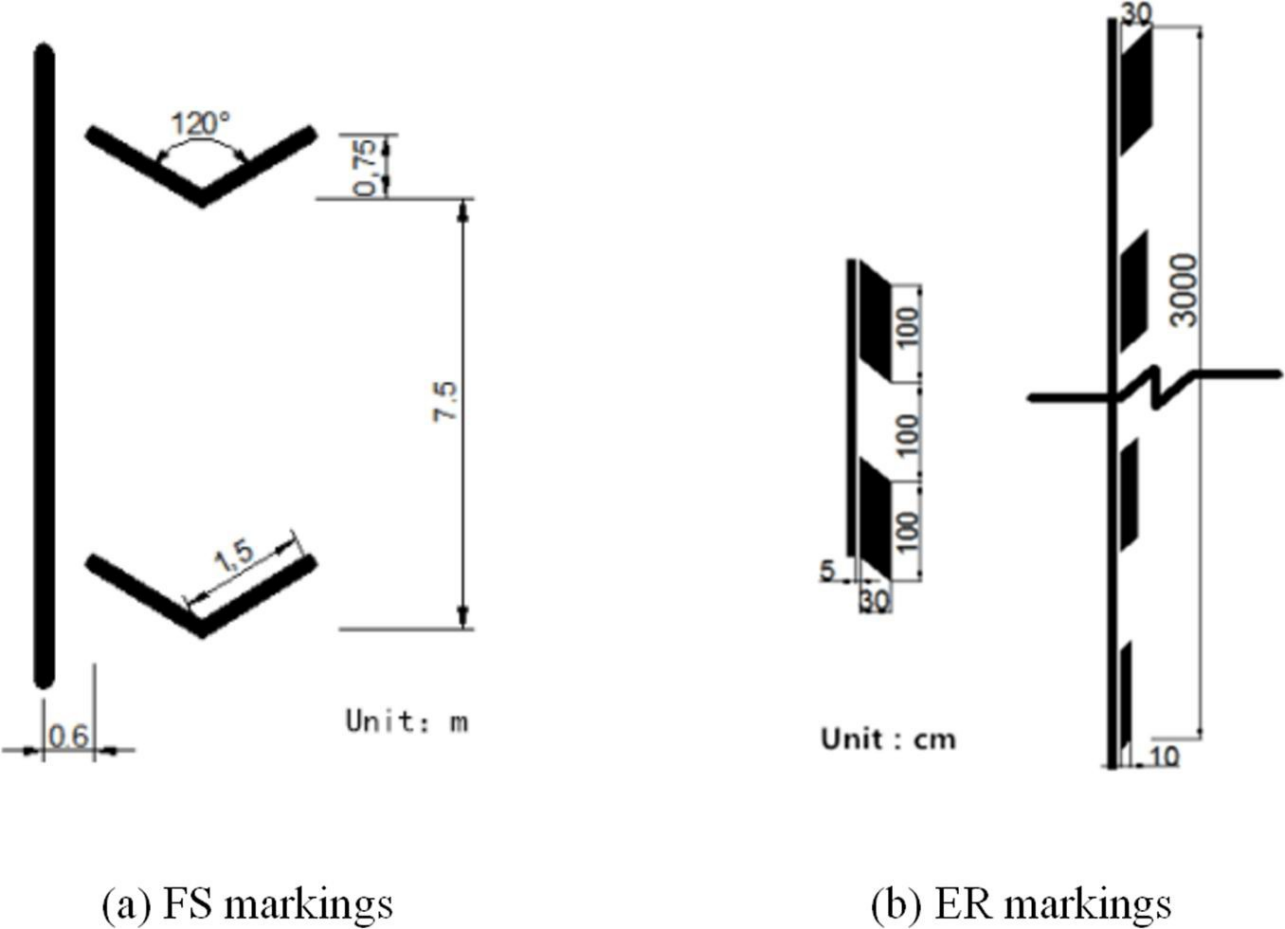
Dimensions of fishbone-shaped and edge-rate deceleration marking. (a) FS markings. (b) ER markings.

The design limited speed of experimental road is 120 km/h. However, in consideration of speeding driving, this paper assumed that the maximum speed is 130 km/h. The deceleration markings were set in front of the Xiaohe Bridge with a limited speed of 90 km/h. Once the vehicle passed the road of deceleration markings, its speed needed to reduce to the range of limited speed. Hence the length of deceleration markings was set as 200 meter. Meanwhile, two types of markings were selected for evaluation in this study (FS and ER markings). The dimensions of the deceleration markings are based on Technical Standard of Highway Engineering [29]. The FS markings were white, 1.5-m wide and the width of single markings were 30-cm with an angle of 120° between the sides of the marking (Fig 2A). The spacing between the markings is initially 7.5 m and then gradually decreases to 5.1 m after 15 markings. The ER markings were diamond-shaped blocks that were set parallel to the road edge. The distance between the blocks was 100 cm, the length of the diamond was 100 cm, and its width ranged from 10 cm to 30 cm. The sharp angle of the diamond was 45 degrees (Fig 2B). The design dimensions of the ER markings were based on the guide on Road Traffic Signs and Markings [29, 30].

### Construction of experimental scenarios

**Fig 3:**
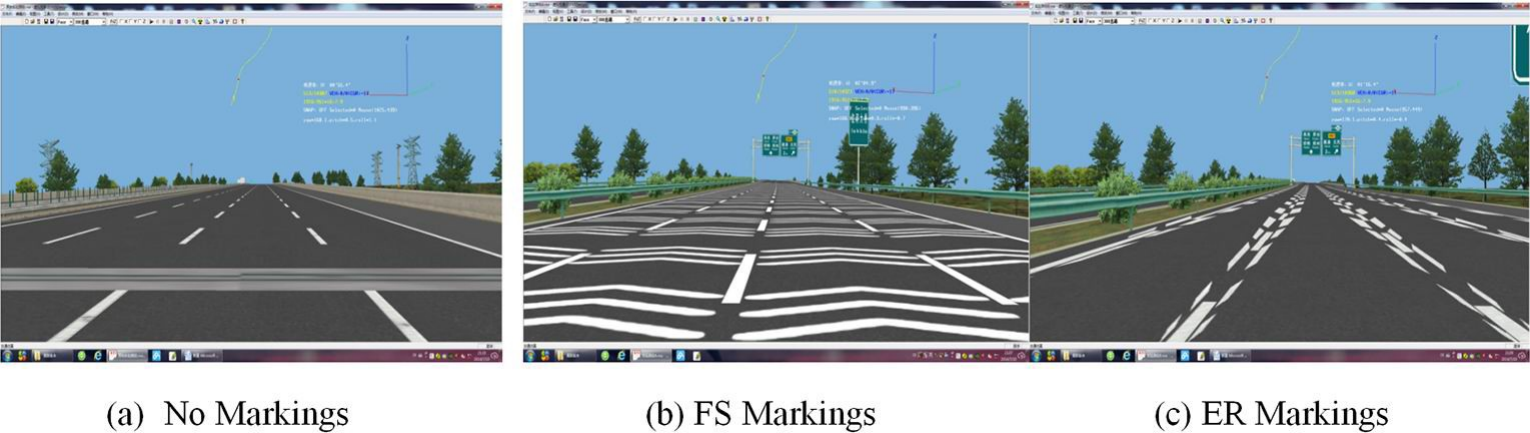
Experimental scenarios for deceleration markings used in the study. (a)No markings. (b) FS markings. (c) ER markings.

The experimental scenarios were constructed using Multigen Creator, a rapid modeling software. The software allows the user to establish road scenarios and gathers relevant experimental data quickly. Then, the software automatically generates three-dimensional models. The simulated section of Shijiazhuang-Anyang Expressway included basic road geometric characteristics, Xiaohe Bridge, Huangshi Interchange, and Huangshi toll station. The simulated section also included standard lane markings, guide signs, and various road landscapes, such trees and scenery. A pavement with good conditions and sunny weather were adopted in the scenarios. Based on freeway construction specifications and relevant signs/markings guidelines of China, the deceleration markings were constructed. In the experiment, there are three experimental scenarios shown in Fig 3, and the type of markings is the independent variables (No markings, FS markings, and ER markings).

## Data collection

### Experimental process

**Fig 4:**
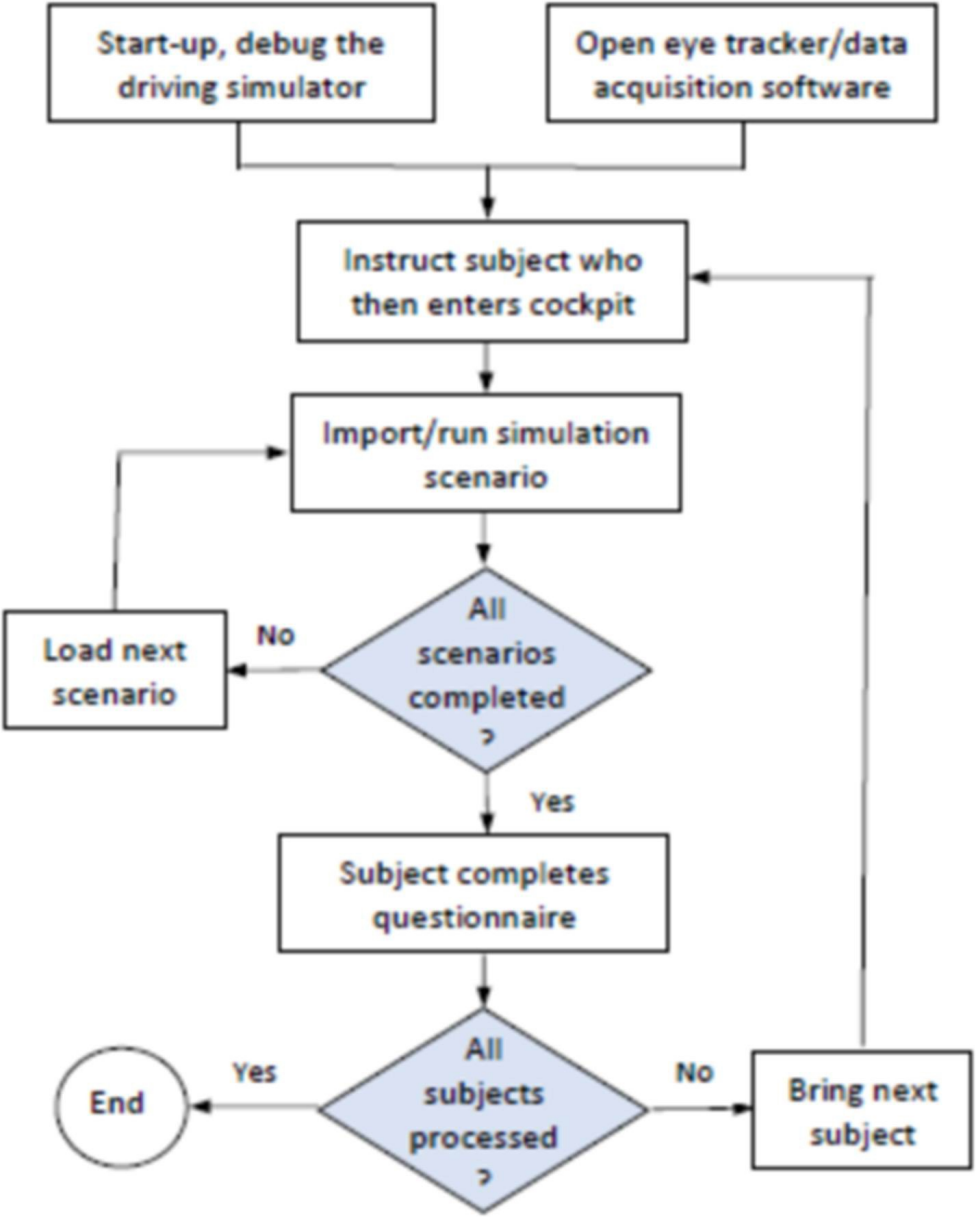
Procedures of the driving simulator experiments.

Before the start of the experiments, a non-markings freeway road that was created on the driving simulator was used to train each subject driving for 3 to 5 minutes in the simulator for the familiarized operation the vehicle. The subjects were then instructed about the driving operations and the limited speed of simulator. To ensure that the subjects would drive according to their normal and real-driving habits, the training experiment was designed as a double-blind trial, where the subjects were not informed about the purpose of the experiment or experimental scenarios. By using the within-subject design, each subject drove the three experimental scenarios (no markings, FS markings and ER markings) under three traffic flow levels (low, medium, high). The total duration time of driving in the simulator was 54 minutes. Because of 9 conditions of driving (3markings types*3 traffic flows), the random Latin order design was used to balance the order effect towards the driver’s expectation. For each condition, the drivers were required to drive around 6 minutes (6km length each). In order to eliminate driving fatigue, the drivers were instructed to have 5 minutes rest after three conditions driving, which in total had 15 minutes rest through the whole driving experiment. Meanwhile, light cookies and soft drinks were provided for the drivers during the period of rest. At the end of the experiment, each subject completed a questionnaire regarding his/her subjective assessments after driving. In fact, the whole duration of the driving experiment for each participant was roughly about 90–100 minutes. The specific procedures are presented in Fig 4.

### Data collection process

For the purpose of this experiment, the parameters of eye movement and the vehicle’s movement condition were collected such as vehicle speed, perceived distance, pupil diameter and brake pedal depth. The characterization parameter of vehicle movement condition is generated in the simulation system directly. Each parameter of driving simulator would be adjusted before the experiment starts and the acquiring frequency of driving simulator would be set as 30ms. The experimental data of relevant vehicles can be exported as required by the export interface of relevant data in the system. By utilizing Excel and Stata to process and analyze data, the file formats of data exported by driving simulator could be transformed from ".pth" and ".rep" to ".xls".

**Fig 5:**
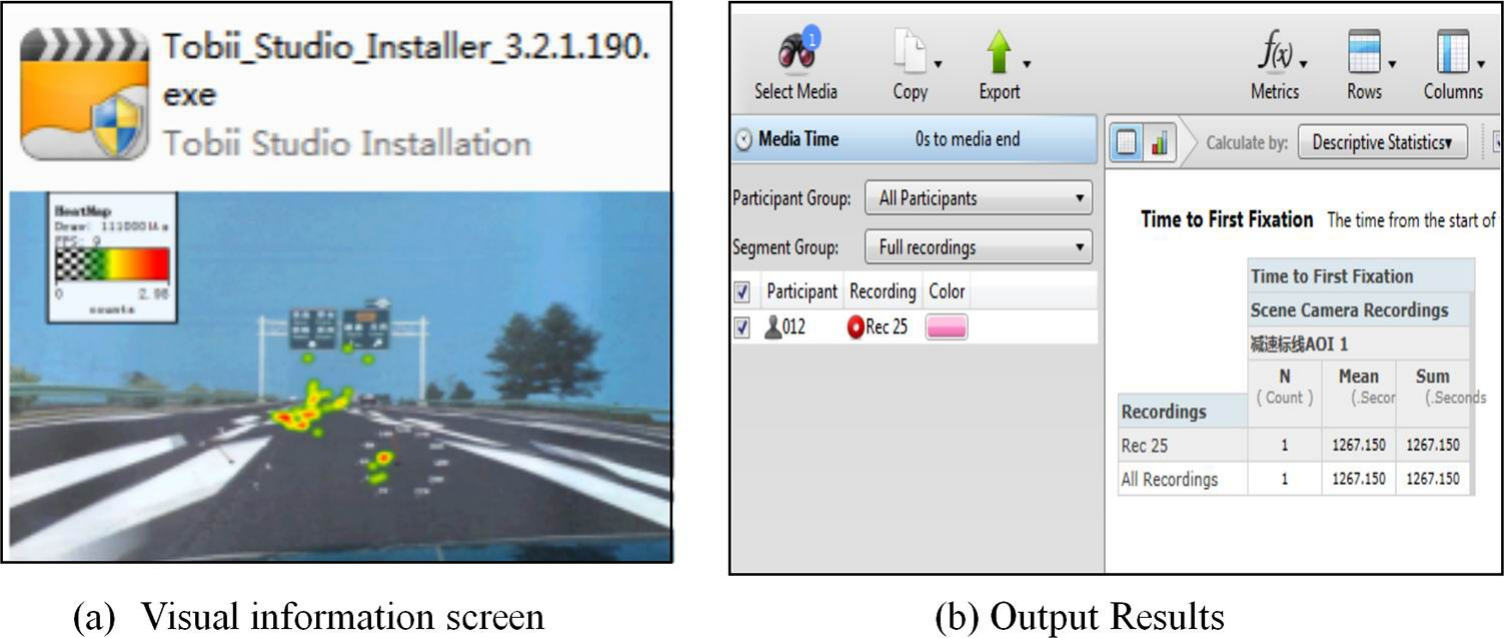
Visual information screen and output results in Tobii Studio. (a)Visual information screen. (b) Output results.

The Tobii X2-30 data were generated in the Tobii Studio, with 30Hz sampling frequency (standard deviation is about 3Hz) and 35 to 67 ms system delay. The data generated by the eye tracker included documents of Event and Samples and video files of avi as well as videos from the four channels of the video monitoring system. Tobii Studio can not only supervise and record the subject’s eye movement behavior in real time, but also support the creation and statistics of visual data for video clips. The raw data were then exported in Excel format using the interception and conversion process (Fig 5).

## Analysis of results

Except for some subjects who have no eye movement data and driving simulator data, or those whose eye movement time doesn’t correspond with driving simulator time, the rest of subjects’ data from driving simulator and that from eye tracker were integrated and corresponded respectively in chronological order. After effective data filtration, the experimental data of 48 drivers were used for final analysis. Repeated ANOVA was applied to the within-subject design.

## Effect of contrast on vehicle speed

**Fig 6:**
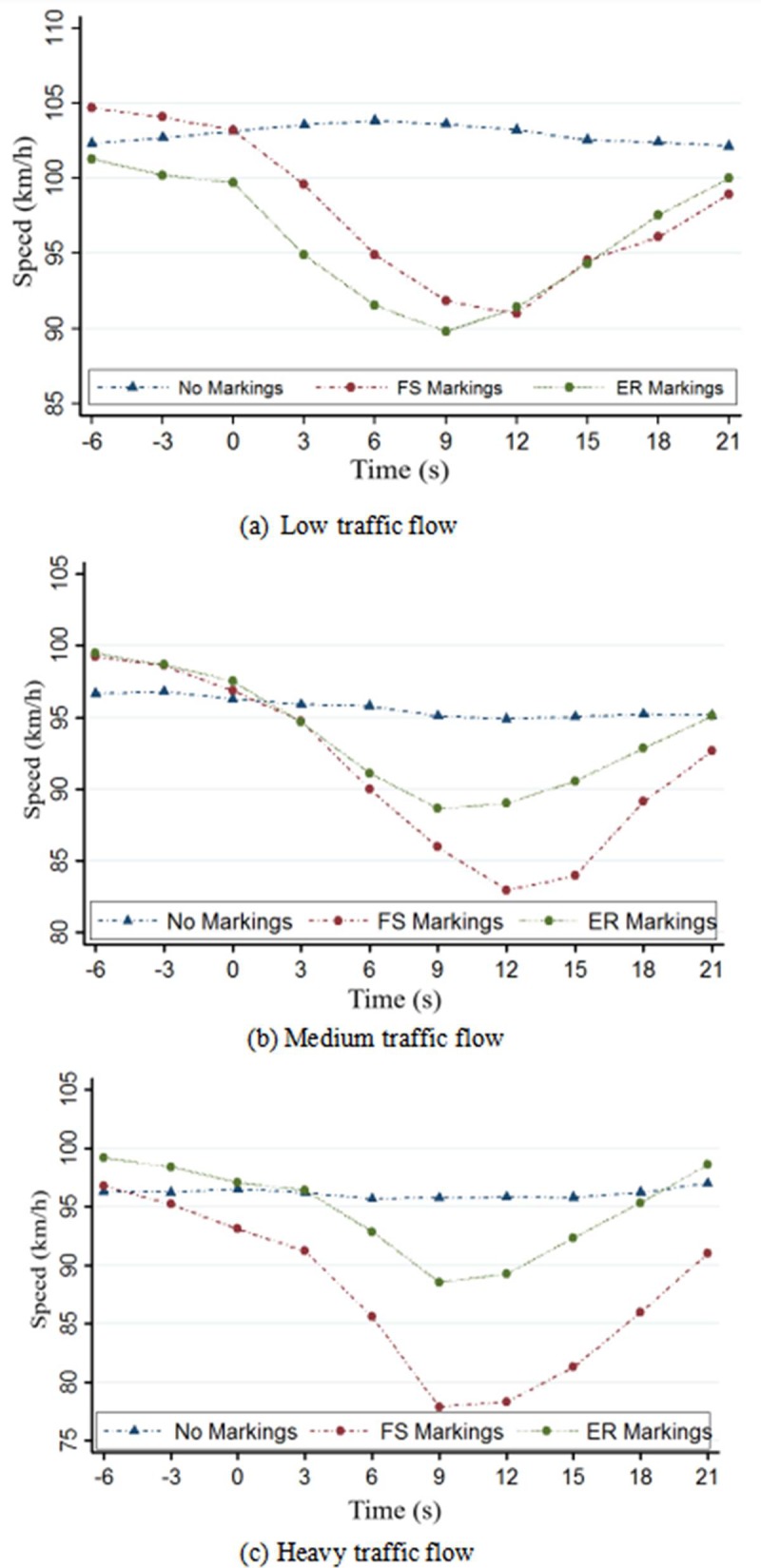
Variation of speed with time at deceleration markings for different traffic flow levels. (a) Low traffic flow. (b) Medium traffic flow. (c) Heavy traffic flow.

In order to analyze the features of speed change in the process of passing the deceleration markings section, we collected ten speeds when (it is defined as "0" o’clock) subjects saw markings at the first sight through the driving experiment. After the data processing of STATA, the curve of speed—time variation around deceleration-markings was presented in the Fig 6.

It can be seen clearly from Fig 6 that drivers controlled their driving speed very well and maintained the speed of passing the deceleration markings section at a reasonable rate in the presence of deceleration markings. The difference of the deceleration effect between the two types of markings (FS and ER) is not so obvious. However, the deceleration effect of FS markings is more distinct particularly under heavy traffic flow. In general, the deceleration effect of FS markings is better with longer deceleration duration, while that of the ER markings is relatively worse, but more smooth.

The speed-time curves of Fig 6 show the deceleration trend of vehicles under three traffic flows during the driving period with three types of markings (no markings, FS, ER). To analyze the deceleration effect, the data were selected in this study from the first time the driver sees the deceleration markings to the end of the deceleration behavior. The change of speed (deceleration) and the condition of deceleration distance during this period were analyzed. After filtering the data using STATA software, the statistics of the speed at the deceleration markings sections were obtained as shown in Table 2. The deceleration rate and deceleration ratio [23] are calculated as

$$a=\frac{v_{1}-v_{2}}{t}$$ (1)

$$r=\frac{v_{2}-v_{1}}{v_{2}}\times 100$$ (2)

where a = deceleration rate (m/s^2^), v1 = initial speed (m/s), v2 = final speed (m/s),

and r = deceleration ratio (%)

**Table 2:** Statistics of the speed of deceleration markings section.

Traffic Flow Level	Scenario Type	Initial Speed (km/h)	Final Speed (km/h)	Difference (km/h)	Deceler. Ratio (%)	Deceler. Time (s)	Deceler. Rate (m/s^2^)	Deceler. Distance (m)
**Low**	**FS**	103.2	91.0	12.3	11.9	9.0	0.378	243
	**ER Markings**	99.7	89.8	9.9	10.0	8.5	0.324	224
**Medium**	**FS**	96.9	83.0	13.9	14.4	10.5	0.68	262
	**ER**	97.51	88.64	8.87	9.10	8.5	0.290	220
**Heavy**	**FS**	93.08	77.89	15.20	16.33	11.5	0.367	273
	**ER**	97.05	88.53	8.53	8.79	9.0	0.263	232

From Table 2, the deceleration effect of FS markings was the best under all three levels of traffic flow, with longer deceleration duration and greater deceleration rate (or greater stimulus of deceleration). Generally, the deceleration rate of both types of markings was slower (much less than 1.8 m/s^2^), which may not cause either a strong deceleration behavior or a dangerous behavior of emergency braking. It can be observed from Fig 5 that the deceleration curve of the FS markings was relatively sharper than the other markings (F(2, 94) = 32.78, P<0.05). In particular, under heavy traffic flow, influenced by nearby vehicles, the subjects passed over the FS markings with greater reduction in speed

### Effect on perceived distance

The perceived distance shows the distance from markings that are firstly perceived by drivers to the point of markings, which reflects the visual effect of markings. The longer the perceived distance is, the more sufficient time for drivers to react and make decisions and stronger capability of controlling speed. By Tobii Studio, we create AOI (Analysis of Interest) of deceleration markings, the first fixation that subjects see AOI of deceleration markings is determined via TFF—the statistical parameter of eye movement. The difference between the current displacement exported from driving simulation and displacement of the starting point of deceleration markings is the perceived distance. The statistics of subjects perceived distance is as shown in Table 3.

**Table 3:** Average perceived distance for the two types of deceleration markings for different traffic flow levels.

Type of Deceleration Markings	Average Perceived Distance (m)		
	Low Traffic Flow	Medium Traffic Flow	Heavy Traffic Flow
**FS**	84.63	81.37	74.22
**ER**	49.13	43.83	41.70
**t (t**_**α/2**_ **= 1.96)**	4.56.	5.48	4.01
**Pr (95% confidence)**	0.004	0.002	0.005
**Significance**	Yes	Yes	Yes

According to the statistics, the average perceived distance of FS markings are 84.63m, 81.37m and 74.22m respectively and the average perceived distance of ER markings are 49.13m, 43.83m and 41.70m respectively. In the three conditions of traffic flow, the perceived distance of FS markings is longer than that of ER markings, which means drivers have more sufficient reaction time and longer deceleration distance when they pass the section of deceleration markings.

The perceived distance reflects markings’ visibility and its capability of attracting attention. Because of the high flash frequency of FS markings and the wide area occupied by markings, the FS markings might attract drivers attention more easily. This indicates that FS markings’visibility and its capability of attracting attention are relatively superior to ER markings. In general, both two deceleration markings are able to attract drivers’attention for a period of time. Meanwhile, these two deceleration markings may not cause drivers’neglect or too much attention, and they are likely to convey the deceleration information to drivers under moderate intensity.

By combining the speed and perceived distance statistics, the following observations can be made:

1. For the FS markings, the vehicle will be ready to decelerate when it is at a distance of 70–90 m from the deceleration line and pass it in a comfortable manner. The deceleration time will last 9–12 s (which corresponds to the 200 m deceleration line. For the ER markings, the corresponding distance and deceleration time are 40–60 m and 8–10 s, respectively. However, it shows little change in the extent of speed. In both cases, when the fixation point becomes away from the range of the deceleration line, the vehicle will accelerate until the speed reaches the normal speed that is designed by the experiment. In comparison, the ER deceleration distance is relatively smaller, which shows that the perceived distance is shorter and its deceleration stimulation is lighter than that of the FS markings.
2. Under the circumstances of the three traffic flow levels, the deceleration effect and visibility of the FS markings are better than that of the ER markings. With the FS markings, the drivers could reduce the speed as soon as possible, and the deceleration time would take longer. For high traffic flows, the deceleration availability would be more obvious. Also, the larger the speed difference, the longer the deceleration time would be.

### Effect on driver behavior

The driving process is the course of circulation of perception, estimate, decision and manipulation. Therefore, in addition to study the deceleration availability from the speed index, it can be analyzed the availability of the deceleration line by comparing the drivers braking times or brake pedal depth in various circumstances of driving. According to the driving behaviors data outputted by the driving simulator, we calculate the average brake pedal depth when the drivers passed the deceleration markings on road (Table 4).

**Table 4:** Statistics of average brake pedal depth in deceleration for different traffic flow levels.

Element	Average Brake Pedal Depth (mm)		
	Low Traffic Flow	Medium Traffic Flow	Heavy Traffic Flow
**FS markings**	0.30	0.30	0.28
**ER markings**	0.23	0.23	0.22
**t (t**_**α/2**_ **= 1.96)**	3.20	2.95	3.35
**Pr (95% confidence level)**	0.0059	0.0100	0.0044
**Is Difference Significant?**	Yes	Yes	Yes

**Fig 7:**
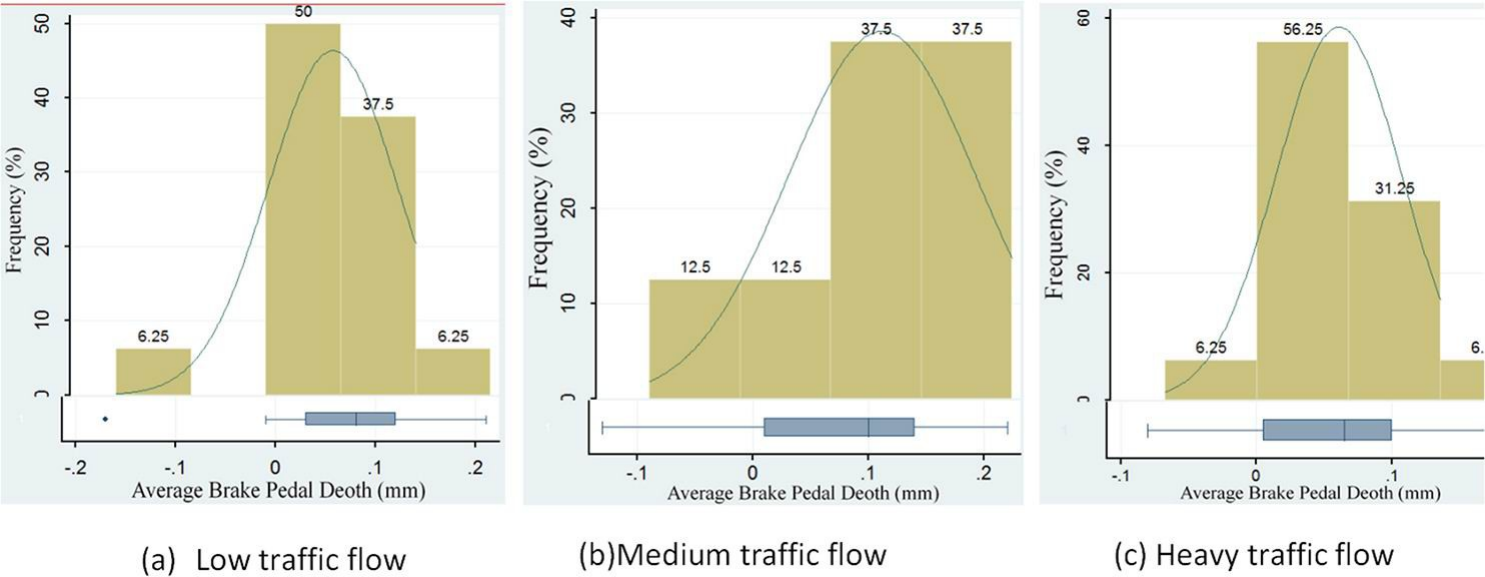
Distribution of the difference in average brake pedal depth for different traffic flow levels. (a)Low traffic flow. (b)Medium traffic flow.(c) Heavy traffic flow.

Seen in the Table 4, it is noted that (under all three traffic levels) the average brake pedal depths of the FS deceleration markings are 0.30 mm, 0.30 mm, 0.28 mm, respectively, which are significantly greater than those of the ER markings (0.23 mm, 0.23 mm, 0.22 mm). This means that when the driver passes the FS markings section, they would be more likely to decelerate the vehicles as compared to pass the ER markings. Clearly, the type of deceleration marking affects the average brake pedal depth. The Pair t-test was used to check whether the difference between the average brake pedal depths of the two types of deceleration markings was significance. The result of the Pair T-test showed that two kinds of deceleration markings have obvious differences in the influence on the pedals average depth under all three traffic flows conditions. Aiming at the difference values of the average brake pedal depth of all subjects, we made the frequency distributions of the average brake pedal depths difference value of the FS and ER markings under different traffic flows (F(2, 94) = 24.53, P,0.05), as shown in Fig 7.

From the distributions of the difference average brake pedal depth shown in Fig 7, it is noted that, under the three traffic flow levels, the difference in the average brake pedal depth of different deceleration markings include both positive and negative values, but most values are on the positive side. This means that when the driver passes the FS marking section, its strength of braking is stronger than that of the ER marking. Using the Pair t-test to test the average brake pedal depth of all drivers under different deceleration markings and compare the difference value distributions, the results showed that the two types of deceleration markings made a significant effect on the average brake pedal depth, while the effect of the FS markings was stronger than the other markings.

### Effect on pupil diameter

The pupil diameter can directly reflect the extent of driver’s tension and the difficulty in performing the task. When the driver’s pupil becomes bigger, it means that the driver maybe in the tension situation or the mental workload is increasing. To analyze the change in driver’s vision workload before and after they see the deceleration markings, the eye tracker was used to record the change of pupil diameter when the driver passes the deceleration marking area and observe their comfort. In this study, ten observation sections of the pupil diameter were selected.

**Fig 8:**
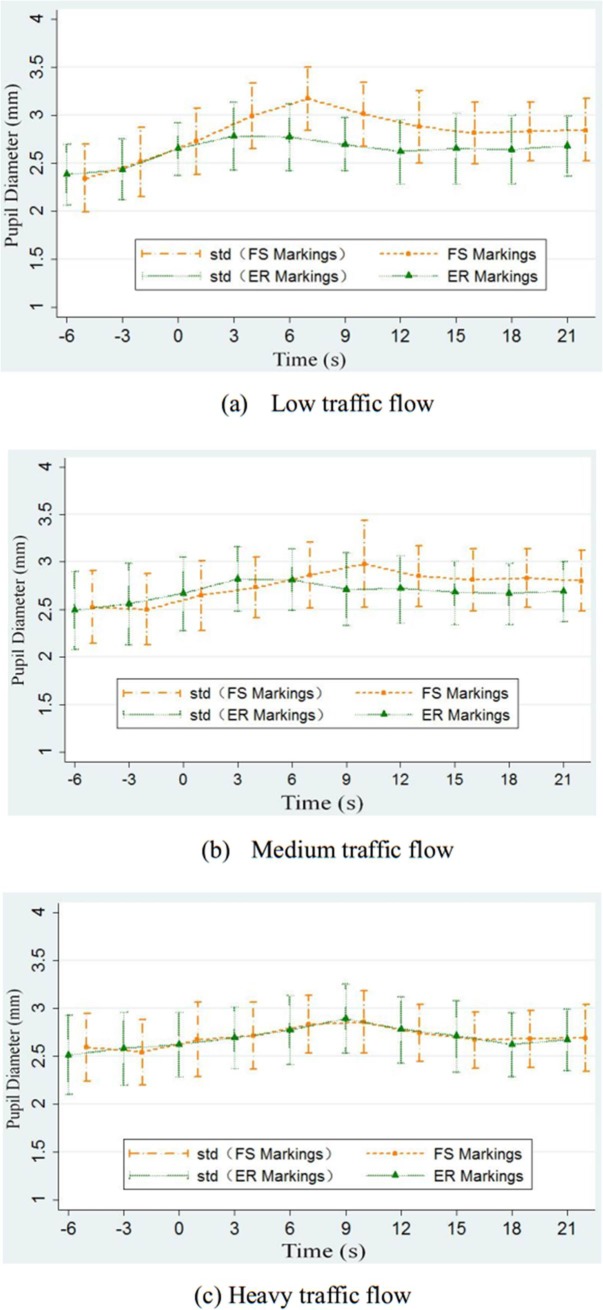
Change of drivers’pupil diameter for different levels of traffic flow. (a)Low traffic flow. (b)Medium traffic flow. (c) Heavy traffic flow.

All the subjects’average data were used in STATA to calculate the pupil diameter’s statistical parameter. The trend of the change in driver’s pupil diameter under three levels of traffic flow is shown (F(2.94) = 14.73, p<0.05) in Fig 8.

From Fig 8, it is noted that when the driver gets close to the deceleration markings, his/her pupil diameter slightly changes. When seeing the deceleration markings, the driver becomes nervous because of the rare information of the road conditions to be sure. To see the road ahead clearly the subject has to pay more attention to it and therefore enlarges the pupil to obtain more traffic information. When the vehicle is closer to the deceleration markings, the driver becomes more comfortable driving on road which in turn may decrease the pupil diameter to. Seen in Fig 8, FS markings could cause driver’s more obvious pupil diameter change, meaning that when the driver passes the FS markings, he or her needs to receive a more intense visual impact and make a more complicated visual mission which can easily cause feelings of tension and fatigue.

### Discussion and guidelines

An experimental design was performed in the current study in order to determine the effectiveness of speed reduction markings. Using a design of driving simulator and a technique of eye-tractor, the researchers analyzed the degree of reduction driving speed, driving behavior (brake depth) and eye-movement parameters (pupils and perceived distance) among two types of reduction speed markings (FS vs ER) as compared to the non-markings on freeway road under three types of traffic flows (low, medium, high). In general speaking, the results were more positive to show that both FS markings and ER markings were more effective than non- markings. While, FS markings show more effectiveness than that of ER markings. These results were in consistent with previous research [6, 7, 8], supporting the fundamental theory of reduction speed markings in a way of giving the drivers’ illusion of perception of acceleration at speed. For example, Gates et al found approximately 1- 4mph reductions in mean speed observed between the before and the after the treatment of transverse pavement markings. While, Drakopoulos and Vergou evaluated the effect of a converging chevron treatment and showed reductions in the mean and 85^th^ percentile speeds of 15–17mph after installation of the chevron markings.

In terms of eye-movement analysis, the results have found that driver’s pupil diameter slightly changes when the driver becomes close to the deceleration markings. The changes for the FS markings ranges from 0.4 mm to 0.8 mm and that for the ER markings ranges from 0.3 mm to 0.4 mm. Clearly, the FS markings causes a substantial change in the pupil compared with the ER markings, indicating that the FS markings causes more visual cognitive effect on the drivers. More specifically, for the FS markings, the driver begins to decelerate at a distance of 70–90 m from the deceleration line, and the deceleration time lasts for 9 to 12 s. For the ER markings, the driver begins to decelerate at a distance of 70–90 m from the deceleration line, and the deceleration time lasts for 8 to 10 s. The visual effect of the ER markings is slightly less than that of the FS markings. These results were well in line with the claims of previous research suggesting that visual information processing of hazard level (reduction speed markings) is remarkable effective and utilized to guide the eyes towards potential illusion hazards [23]. The research also used the driving simulator to compare the deceleration effect and driving behavior between FS and ER marking under three traffic flow levels. Specifically, from low traffic flow to heavy traffic flow, the speed differences of the FS markings are 12.3 km/h, 13.9 km/h, and 15.2 km/h, respectively, and the average petal depths are 0.30 mm, 0.30 mm, and 0.28 mm, respectively. As a result, the FS markings are slightly suitable for roads with complex alignments, such as long and steep downhill sections and sharp horizontal curves. However, the sudden deceleration stimulation and the rapidly changing retardation curve of the FS markings may produce uncomfortable feelings to the driver, such as dizziness. The speed differences of the ER markings for three traffic flow levels from low to high are 9.9 km/h, 8.87km/h, and 8.53 km/h, and the average brake petal depths are 0.23 mm, 0.23 mm, and 0.22 mm, respectively. Under heavy traffic flow, the deceleration effect of the ER markings is modest. Therefore, the ER markings are suitable for gentle highway alignments and low to medium traffic flow levels.

Based on the current results, a reference guide for the use of the two types of deceleration markings under different road environments and traffic flow levels are suggested and presented in Table 5. As noted, both types of deceleration markings are appropriate for the combinations of low traffic-gentle alignments and heavy traffic-complex alignment. However, for gentle alignments, the ER type is recommended for medium to medium traffic and for complex alignments the FS type is recommended for low to medium.

**Table 5:** Guidelines for selection of different types of deceleration markings.

Type of Road Alignment	Traffic flow Level		
	Low	Medium	Heavy
**Gentle**	ER	ER	ER
**Complex**	FS	FS	ER or FS

## Conclusions

This paper has presented an evaluation of the effectiveness of two types of deceleration markings for freeways under different traffic flow levels. Using a driving simulator and eye tracker equipment, six simulation scenarios of freeway deceleration markings were developed and data were collected regarding numerous performance measures, including vehicle speed, perceived distance, and average brake pedal depth. Based on the analysis of the deceleration availability, visual effect, and mental effect, the following comments are offered:

1. When the driver becomes close to the deceleration markings, his/her pupil diameter slightly changes. The change for the FS markings ranges from 0.4 mm to 0.8 mm and that for the ER markings ranges from 0.3 mm to 0.4 mm. Clearly, the FS markings causes a substantial change in the pupil compared with the ER markings, indicating that the FS markings causes more visual and in turn mental effect on the drivers.
2. For both types of markings, the vehicle begins to decelerate at a distance of 70–90 m from the deceleration line. However, for the FS marking the deceleration time is longer than that of the ER marking by about 205. The visual effect of the ER marking is worse than that of the FS markings.
3. Under the three traffic levels, the deceleration effect of the FS marking is generally better for complex highway alignments, such as long and steep downhill sections and sharp horizontal curves. However, the sudden deceleration stimulation and rapidly changing retardation curve of the FS marking may produce uncomfortable feelings to the driver, such as dizziness. Therefore, the FS marking is recommended only for roads with low to medium traffic. On the other hand, under all traffic levels, the deceleration effect of the ER marking is generally better for gentle highway alignments.

In conclusion, the results of the analysis suggest that the experimental pavement markings treatment was effective at reducing speeds on the freeway road. However, to generalize the results to all the driving conditions should be carefully reconsidered in various scenarios because the freeway road used in the simulated experiment was the one type of freeway in China. Further research is needed to examine the transferability of these findings for different situation such as land section, road section, other environmental conditions, or a combination of various variables between different driving experience drivers. Besides, to examine the real-word driving effect by using the real world driving design could complement this study in future research.

As like in many experimental studies, there are limitations to this study that have to be mentioned. For a comparison of the reduction speed effect and visual cognitive effect, in a within-subject design, a sample size of 60 drivers appears acceptable. Still, a larger sample size with more heterogeneous age would be desirable. Results have to be treated with caution when generalizing the findings from this study to the overall driving road sections and all driving conditions.
